# Supplementary material for: Hierarchical composition of reliable recombinase logic devices
Source: Nat Commun. 2019 Jan 28;10:456. doi: 10.1038/s41467-019-08391-y (PMC6349923; doi:10.1038/s41467-019-08391-y)
Supplement: Supplementary file 3 — Reporting Summary [file 41467_2019_8391_MOESM3_ESM.pdf]

Supplementary Information:
Hierarchical composition of reliable recombinase logic devices

Guiziou et al.

These supplementary materials contain:

- -Supplementary Figures S1 to S11.
- -Supplementary table 1.

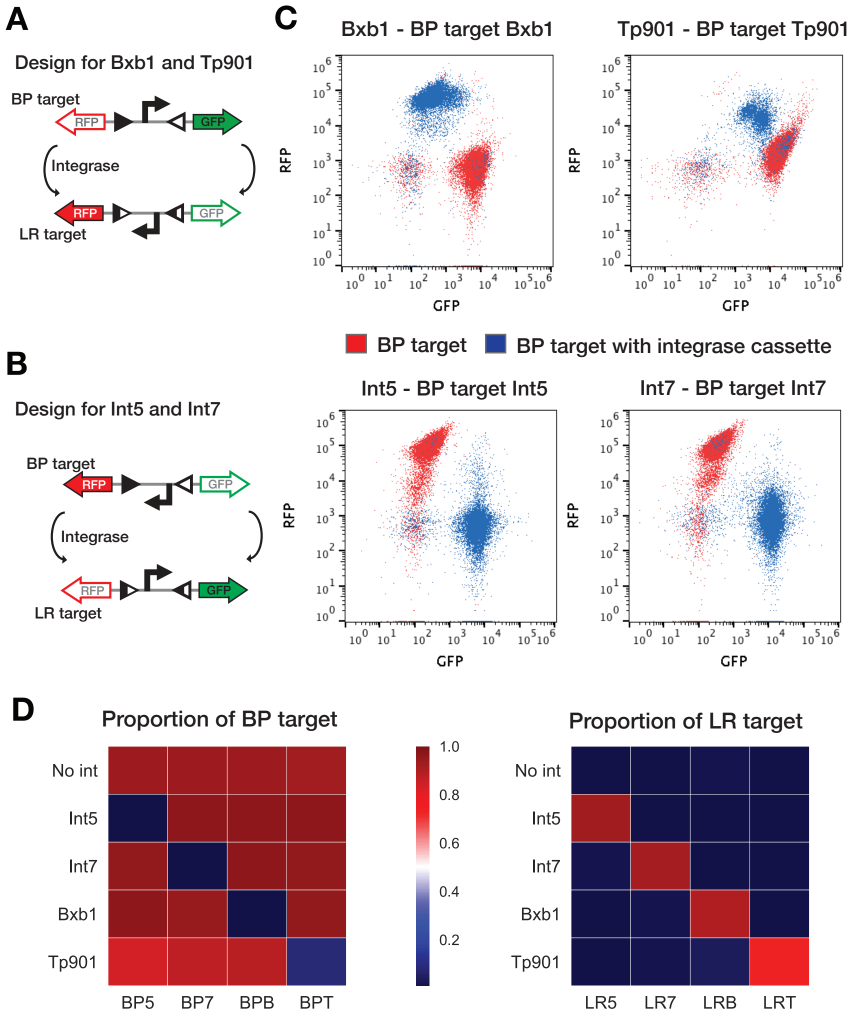

Supplementary Figure 1: **Characterization and orthogonality of 4 serine integrases used in this study.** (A) and (B): Design of BP targets for the integrases. (A) For Bxb1 and Tp901 integrase, in presence of the integrase, gene expression switches from GFP to RFP via promoter inversion (B) for Int5 and Int7, gene expression switches from RFP to GFP. (C) Characterization of each integrase via co-transformation with BP targets. The graphs correspond to the density plots of flow-cytometer experiments with GFP over RFP fluorescence intensity in arbitrary unit. The red dots are *E. coli* strains with BP targets and the blue dots are *E. coli* co-transformed with BP targets and corresponding integrase cassettes. Cells are grown overnight in LB, and with the corresponding inducers for the expression of integrases. (D) Heatmaps of the proportion of BP target (left side) and LR target (right side) in the population of bacteria measured by flow-cytometer. For both heatmaps, each square corresponds to a co-transformation of one integrase cassette or none (labeled in y axis) with one BP target (labeled in x axis, BP5 for Int5, BP7 for Int7, BPB for Bxb1 integrase and BPT for Tp901 integrase).

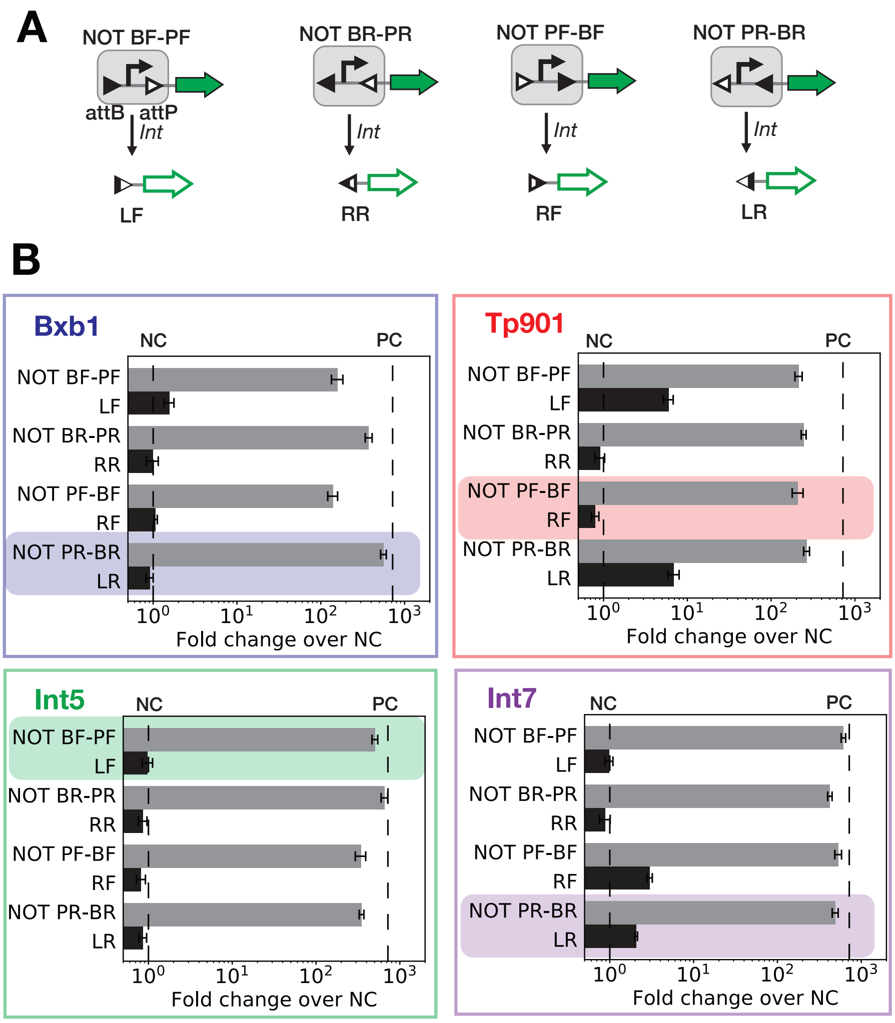

Supplementary Figure 2: **Bar graphs detailing fold changes for the different NOT elements.** (A) The 4 possible designs for NOT-elements, with a promoter flanked by integrase sites, and the corresponding constructs after excision mediated by the integrase. Triangles correspond to the integrase sites, attB site in black, attP site in white and attL or attR in black and white. F denotes sites in forward orientation and R for reverse. The gene coding sequence is a superfolder GFP and the promoter is the P7 promoter. (B) Bar graphs correspond to the fold change in mean fluorescence intensity of constructs compared to the negative control. Data were obtained by flow-cytometry measurement using 3 replicates per experiments, from 3 experiments performed on different days. The grey bars correspond to NOT-elements and the black bars for the attL and attR sites resulting from integrase-mediated excision. The dash lines correspond to fold change of the negative control (NC, equal to 1) and the fold change of the positive control (construct with only promoter P7). Error bars represent the standard deviation between the three experiments.The bars surrounded by a colored box correspond to elements that were selected for assembling recombinase logic devices.

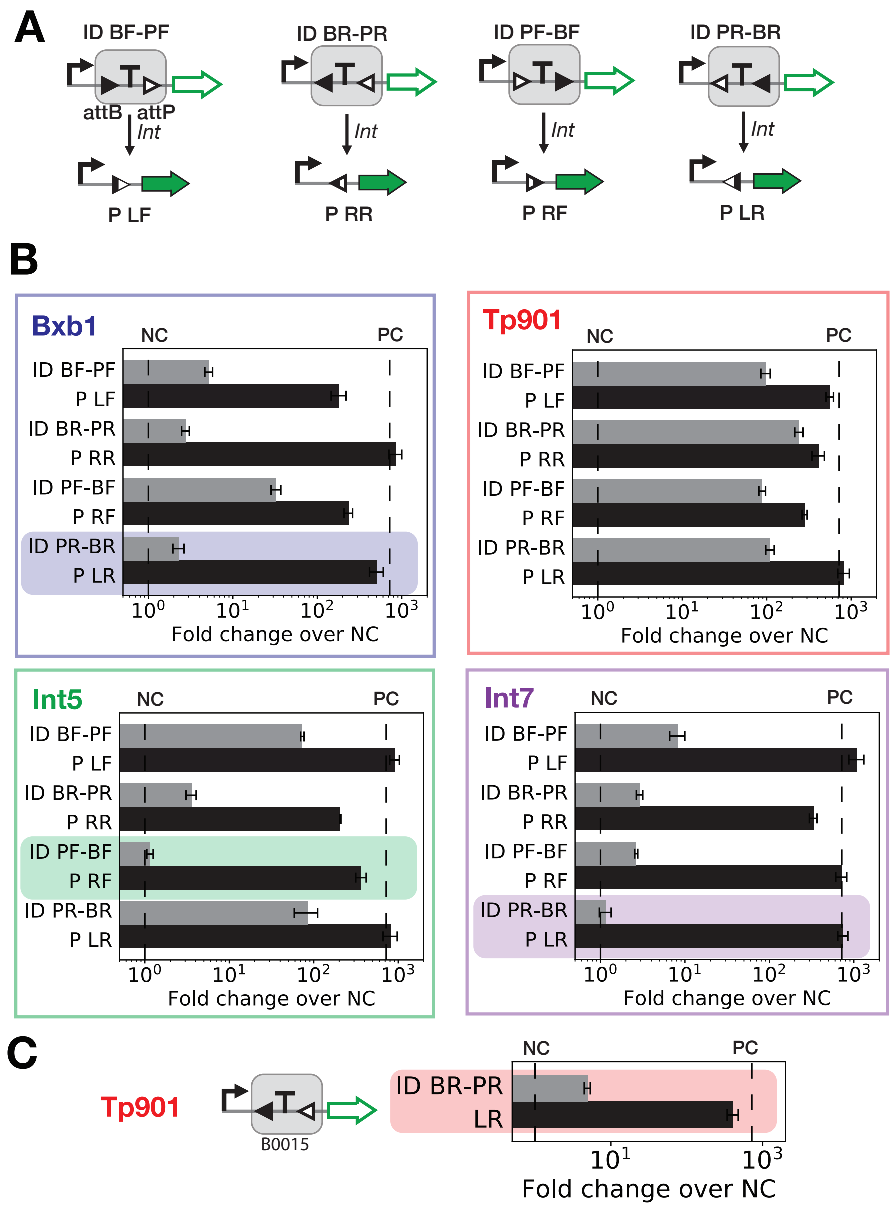

Supplementary Figure 3: **Bar graphs detailing fold changes for the different ID elements.** (A) The 4 possible designs for ID-elements with a terminator flanked by an integrase site pair, and the corresponding constructs after integrase-mediated excision. The gene coding sequence is a superfolder GFP and the promoter, the P7 promoter. (B) and (C) Bar graphs correspond to the fold change of mean of fluorescence intensity compared to the negative control. Data were obtained by flow-cytometry measurement with 3 replicates per experiments, from 3 experiments performed on different days. The grey bars correspond to ID-elements and the black bars for the attL and attR sites resulting from integrase-mediated excision. The dash lines correspond to fold change of the negative control (NC, equal to 1) and the fold change of the positive control (construct with only promoter P7: 650). Error bars represent the standard deviation between the three experiments. The bars surrounded by a colored box correspond to elements that were selected for assembling recombinase logic devices.

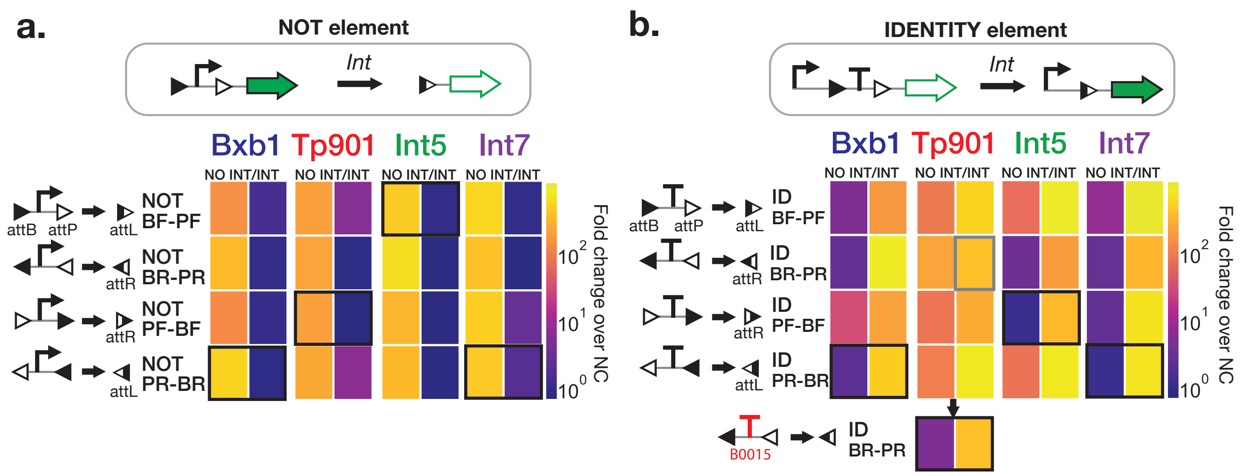

Supplementary Figure 4: **Heatmaps summarizing NOT and ID elements characterization data.** a. NOT elements are composed of a promoter surrounded by integrase sites. In presence of the integrase, the promoter is excised and expression of the output gene is switched from ON to OFF. b. IDENTITY elements are composed of a terminator surrounded by integrase sites. In presence of the integrase, the terminator is excised,and gene expression is switched ON. NOT and IDENTITY elements responding to four integrases (Bxb1, TP901-1, Int5, Int7) were characterized. For each element, four different designs are possible (BF-PF, BR-PR, PF-BP, PR-BR). We measured gene expression before (NO INT) and after switching (INT) by flow cytometry using sfGFP as output (Fig. 2). Cells were grown in LB with appropriate antibiotics for 16 hours at 37C. The data shown in the heatmaps correspond to the fold change in GFP median fluorescence intensity over the negative control (cells with no plasmid). Boxes indicate the construct that was ultimately chosen. A functional TP901-1 ID-element was obtained by replacing the original terminator (lower insert). Data are the mean of 3 experiments performed in 3 different days with 3 replicates per experiment.

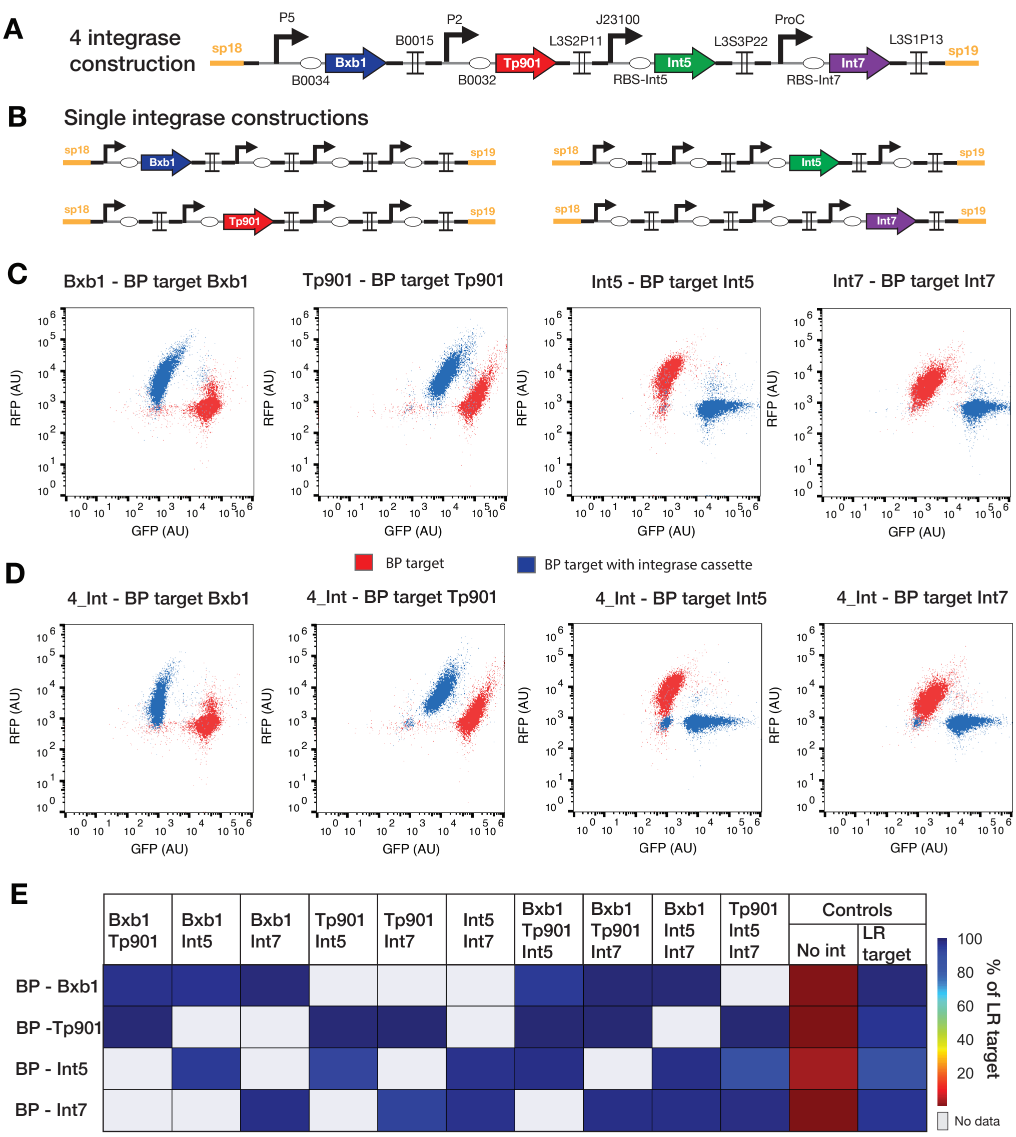

Supplementary Figure 5: **Design and characterization of 16 constitutive integrase expression cassettes.** (A) The 4-integrase cassette is composed of the 4 integrase genes, a different ribosome binding site and promoter for each integrase, terminators to insulate each gene expression cassette and spacers to facilitate gibson assembly cloning. (B) The four single-integrase cassette. (C) Characterization of the four single-integrase cassette and (D) of the four-integrase cassette. Each single-integrase cassette was transformed with its BP target and the four-integrase cassette with each BP target, and after overnight culture, GFP and RFP fluorescence intensities were measured via flow-cytometer. The graphs correspond to density plots of the bacteria population with in x axis GFP fluorescence intensity in A.U. and in y axis RFP fluorescence intensity in A.U. The dots in blue correspond to the BP target with the corresponding integrase cassette and the dot in red correspond to the BP target alone as negative control of the switch. (E) Characterization of the two- and three-integrase cassettes. Each cassette was transformed with BP targets corresponding to the integrase it mediates expression, and after overnight, GFP and RFP fluorescence intensities were measured via flow-cytometer. For each target, on the GFP vs RFP density plot, a gate was defined on the switched population based on the LR target. The percentage of switched population is represented in the heatmap, data represented correspond to the mean of 3 replicates in one experiment. Each square corresponds to one BP target (labeled in y axis) and one integrase cassette (integrase corresponded labeled in x axis). The grey squares correspond to conditions for which no experiment was performed as the integrase corresponding to the BP target is not present in the integrase cassette.

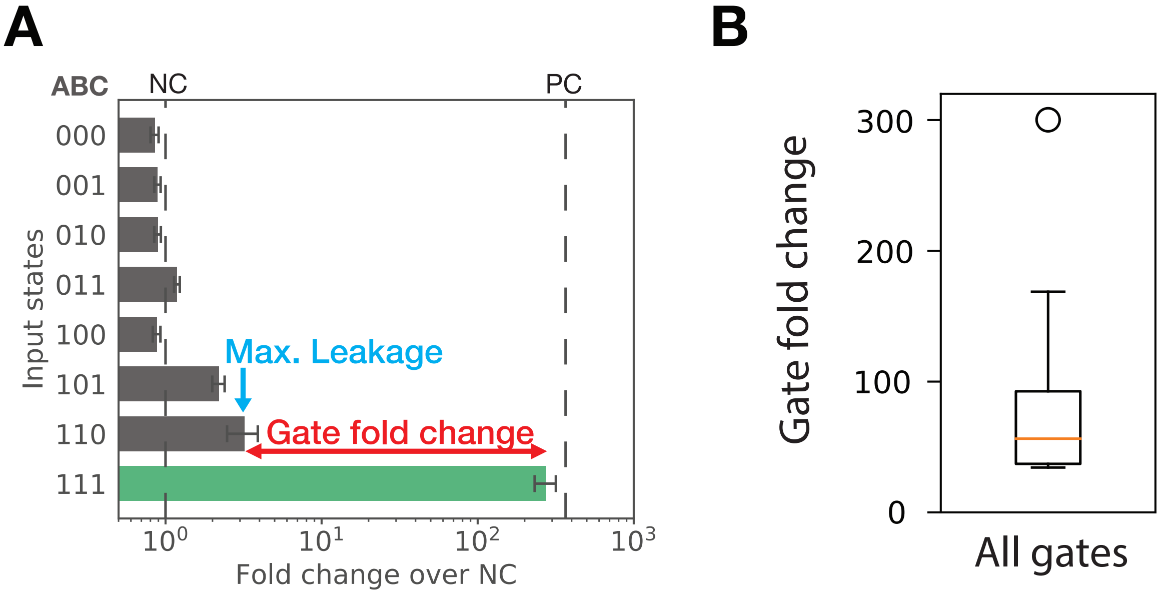

Supplementary Figure 6: **Determination of recombinase logic device performances.** (A) Recombinase logic devices are characterized by a gate fold change and a maximum leakage. The gate fold change corresponds to the fold change between the ON state and the maximum OFF state, equivalent therefore to a minimum fold change. The maximum leakage corresponds to the fold change of the maximum OFF state. (B) Distribution of all 2 to 4-input gate fold changes.

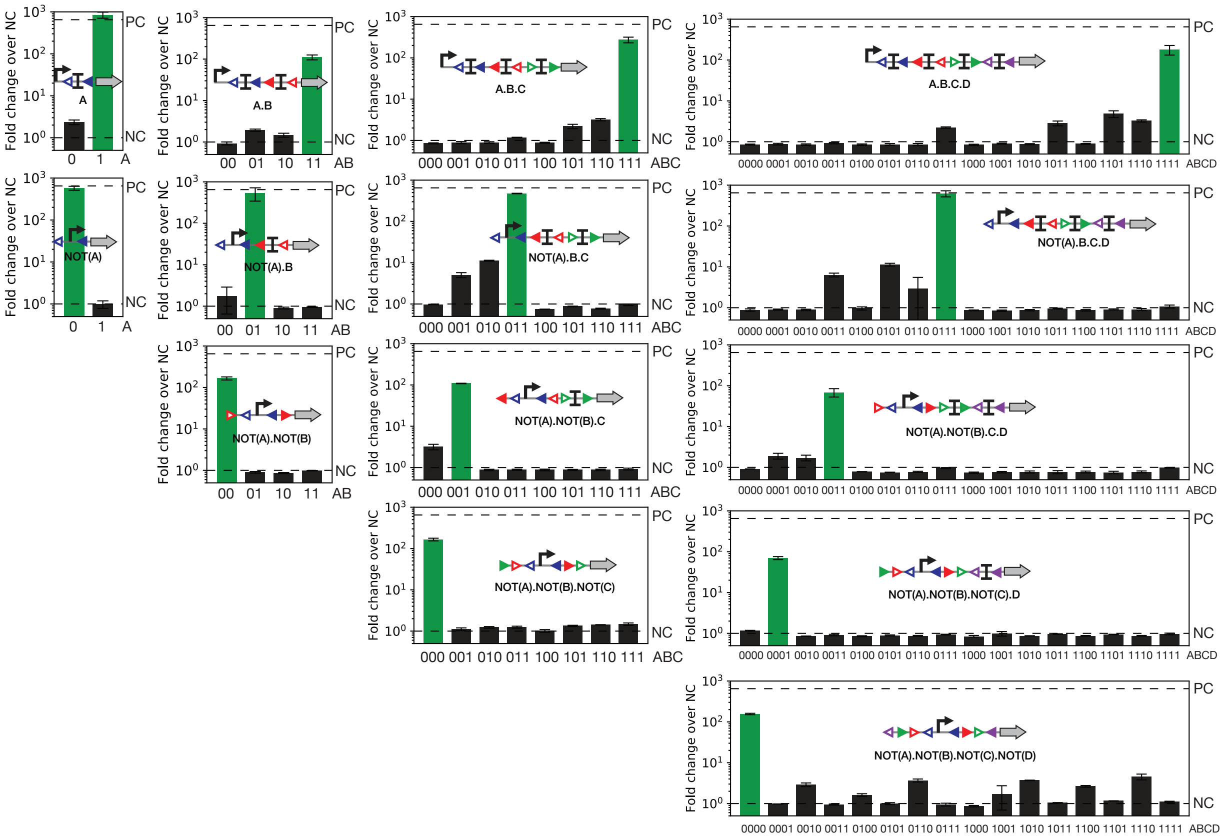

Supplementary Figure 7: **Bar graphs detailing fold changes for the different recombinase logic devices.** We characterized recombinase logic device for each input state by co-transforming each devices with a combinatorial collection of constitutive integrase cassette (Fig. 4). We measured the fluorescence intensity for each state by flow-cytometry ((Fig. 5). For each device, the bar graph corresponds to the fold change of median GFP fluorescence intensity over the negative control. Corresponding input states are on the x-axis (0: no input, 1: input). Green bars correspond to the input states expected to be ON. Fold changes for the negative control (NC, no promoter, Fold Change =1) and positive control (PC, promoter only, Fold Change=650) are represented by dash lines (NC and PC). Data are the mean of 3 experiments performed in 3 different days with 3 replicates per experiment. Error bar: +/- s.d.

# Logic device	# Gate fold change	# Maximum leakage
not(A).B.C	42	11
not(A).B.C.D	55	11
not(A).B.C P6	15	3.2
not(A).B.C.D P6	28	1.9

Supplementary Table 1: Fold changes for recombinase logic devices with reduced transcription input signal. The gate fold change corresponds to the fold change between the ON state and the maximum OFF state, equivalent therefore to a minimum fold change. The maximum leakage corresponds to the fold change of the maximum OFF state.

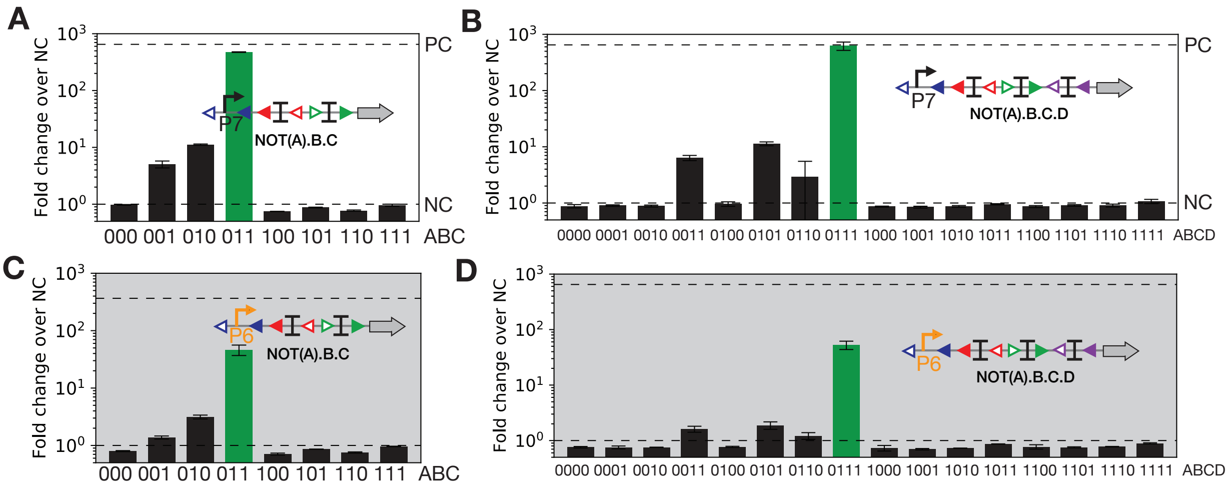

Supplementary Figure 8: **Recombinase device tuning through change in transcription input signal.** We tuned the 2 devices with the highest background level by changing the promoter from P7 to P6. The characterization of the original devices not(A).B.C and not(A).B.C.D with the P7 promoter are represented in (A) and (B) respectively. (C) (D) Characterization of the devices with a weaker transcription input signal (P6 instead of P7). Error bar: +/- s.d.

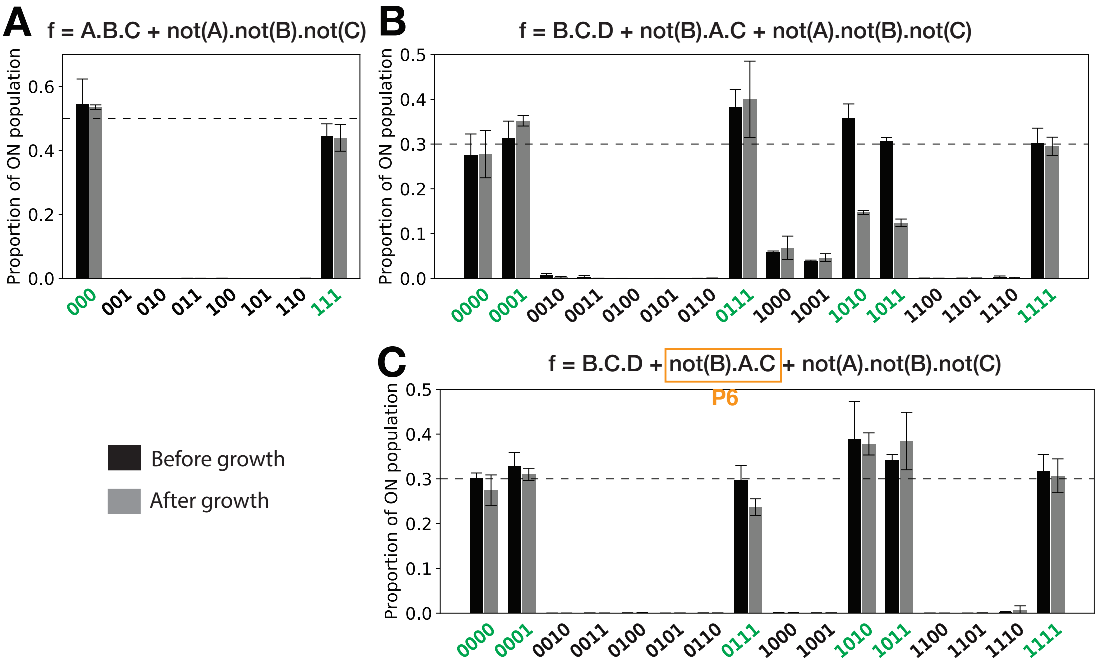

Supplementary Figure 9: **Measurement of the proportions between the different strains of a multicellular system at different time points.** Proportion of ON cells in the total co-culture for each cell. Before (in black) and after overnight growth (in grey), single-cell fluorescence measurement of the co-culture is performed using flow-cytometer. Then, the proportion of cells expressing GFP is defined and plotted. X-axis correspond to the input state simulated by the analysed co-culture. Represented proportions are the mean of 2 experiments and the error bars correspond to the standard deviation between the two experiments.

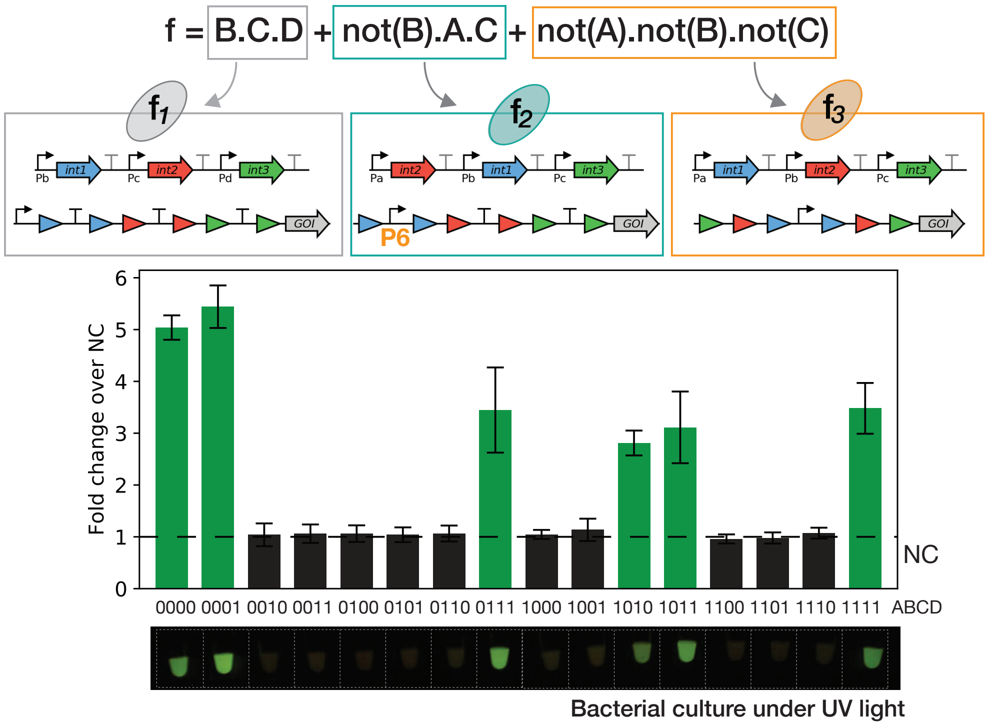

Supplementary Figure 10: A 4-input multicellular logic system using a device tuned by changing the transcription input signal. Characterization of a multicellular logic system for the implementation of the logic function in Figure 3.C (f=B.C.D+not(A).A.C+not(A).not(B).not(C)) using the not(A).B.C logic device with P6 promoter. To prototype this logic system for each input state, we mixed the two strains containing the recombinase logic device and different constitutive integrase cassette corresponding to the different input states. After overnight growth, we measured the bulk fluorescence intensity of the whole-population using a plate reader. Bar graphs corresponds to the fold change in GFP median fluorescence intensity over the negative control. Data are from two experiments performed in different days with three replicates per experiment. Error bars: +/-SD. The photograph correspond to three co-culture replicates for each state centrifuged together, resuspended in 20 µL and observed under a UV light.

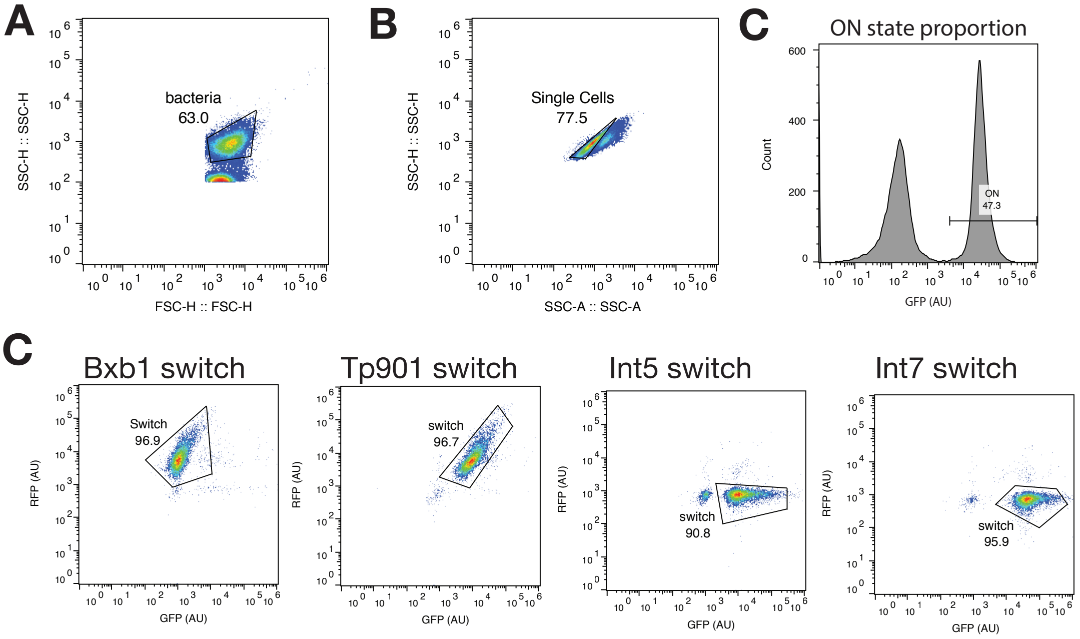

Supplementary Figure 11: **Flow-cytometer gating strategy.** (A) For all experiments, a gate was designed based on forward and side scatter graphs to remove debris from the analysis. (B) For multicellular experiments, from bacteria events, a gate was designed based on side scatter high and area graphs to select single cell events. (C) To determine the proportion of ON state in the figure S9, histograms of GFP fluorescent intensity (BL1:H) on single cell events were plotted and a gate was determine on the events with more than 4200 AU corresponding to the ON states. (D) For the characterization of integrase cassette in Figure S1, the proportion of switched bacteria was obtained with a gate specific to each integrase on GFP over RFP graphs (BL1:H over YL2:H).
