# Supplementary material for: Hierarchical composition of reliable recombinase logic devices
Source: Nat Commun. 2019 Jan 28;10:456. doi: 10.1038/s41467-019-08391-y (PMC6349923; doi:10.1038/s41467-019-08391-y)
Supplement: Supplementary file 6 — Description of Additional Supplementary Files [file 41467_2019_8391_MOESM6_ESM.docx]

**Title:** Supplementary Data 1 – DNA Sequences

**Description:** DNA sequences for NOT and ID elements, recombinase logic devices, and integrase expression cassettes.
